# In Vitro Anti-Diabetic Activities and UHPLC-ESI-MS/MS Profile of *Muntingia calabura* Leaves Extract

**DOI:** 10.3390/molecules27010287

**Published:** 2022-01-04

**Authors:** Nur Khaleeda Zulaikha Zolkeflee, Nurul Shazini Ramli, Azrina Azlan, Faridah Abas

**Affiliations:** 1 Natural Medicines and Products Research Laboratory, Institute of Bioscience, Universiti Putra Malaysia, Serdang 43400, Malaysia; khaleeda_zulaikha@yahoo.com; 2 Department of Food Science, Faculty of Food Science and Technology, Universiti Putra Malaysia, Serdang 43400, Malaysia; shazini@upm.edu.my; 3 Department of Nutrition and Dietetics, Faculty of Medicine and Health Sciences, Universiti Putra Malaysia, Serdang 43400, Malaysia; azrinaaz@upm.edu.my

**Keywords:** *Muntingia calabura*, α-glucosidase inhibitor, α-amylase inhibitor, LCMS identification, absolute quantification

## Abstract

Anti-diabetic compounds from natural sources are now being preferred to prevent or treat diabetes due to adverse effects of synthetic drugs. The decoction of *Muntingia calabura* leaves was traditionally consumed for diabetes treatment. However, there has not been any published data currently available on the processing effects on this plant’s biological activity and phytochemical profile. Therefore, this study aims to evaluate the effect of three drying methods (freeze-drying (FD), air-drying (AD), and oven-drying (OD)) and ethanol:water ratios (0, 50, and 100%) on in vitro anti-diabetic activities of *M. calabura* leaves. In addition, an ultrahigh-performance-liquid chromatography–electrospray ionization tandem mass spectrometry (UHPLC-ESI-MS/MS) method was used to characterize the metabolites in the active extract. The FD *M. calabura* leaves, extracted with 50% ethanol, is the most active extract that exhibits a high α-glucosidase and α-amylase inhibitory activities with IC_50_ values of 0.46 ± 0.05 and 26.39 ± 3.93 µg/mL, respectively. Sixty-one compounds were tentatively identified by using UHPLC-ESI-MS/MS from the most active extract. Quantitative analysis, by using UHPLC, revealed that geniposide, daidzein, quercitrin, 6-hydroxyflavanone, kaempferol, and formononetin were predominant compounds identified from the active extract. The results have laid down preliminary steps toward developing *M. calabura* leaves extract as a potential source of bioactive compounds for diabetic treatment.

## 1. Introduction

Diabetes mellitus (DM) is a chronic metabolic disorder and an inappropriate hyperglycemia due to either insulin secretion deficiency or a combination of insulin resistance and inadequate insulin secretion to compensate blood glucose levels [1]. While the type 1 is usually associated with the destruction of pancreatic islet β-cell by the autoimmune process, the type 2 diabetes involves a combination of insulin resistance and defective compensatory insulin secretion [2]. In Malaysia, one in five adults, or 18.3% of the population, is estimated to suffer from the implications of DM in 2019, and these numbers are expected to grow, as a spike has been observed from 13.4% diabetes prevalence in a 2015 report [3]. There are six major drug classes that manage hyperglycemia in DM patients, including biguanides (i.e., metformin), thiazolidinediones (i.e., pioglitazone), sulfonylureas (i.e., glimepiride), meglitinides (i.e., repaglinide), dipeptidyl peptidase IV inhibitors (i.e., sitagliptin), and α-glucosidase inhibitors (i.e., acarbose) [1,4,5,6,7]. However, the naturally occurring anti-diabetic compounds are now gaining popularity as an efficient and wholesome approach toward diabetes management as an alternative compared to synthetic drugs that cause toxic side effects such as hepatic disorders and other negative gastrointestinal symptoms after the consumption over period of time [8,9].

*Muntingia calabura* L. is one of those many traditional medicinal plants that is known to control blood glucose levels. It is extracted by steeping the dried leaves in hot water [10]. These leaves are also known to cure gastric ulcers, swelling of the prostate gland, and helps in alleviating headache and cold symptoms [11]. *M. calabura*, originating from the family of Muntingiaceae, commonly known as “ceri hutan” in Malaysia, is widely cultivated in the warmer areas of Asia, including Malaysia, Indonesia, Philippines, and tropical America. For its high adaptability to strive, even in poor soil conditions including acidic or alkaline, *M. calabura* is now the most commonly found roadside plants in Malaysia [12]. Several studies have demonstrated the positive attributes of *M. calabura* and its influence against gastric damages, bacterial and viral infections, hypoglycemic and analgesic effects [10,12,13,14]. *M. calabura* mainly consists of several compounds, such as triterpenoids (lupenone); flavonoids (5,7-dihydroxyflavanone, 7-hydroxyflavanone, 5-hydroxy-3,7,8-trimethoxyflavone, 5′-hydroxy-7,8,3′,4′-tetramethoxyflavan, 3,5-dihydroxy-7,4′-dimethoxyflavone); phytosterols (β-sitostenone and β-sitosterol), and chalcones derivatives (2′,4′-dihydroxydihydrochalcone, 2′,4′-dihydroxychalcone, and isoliquiritigenin) [15,16,17]. Several studies have compiled the health benefits of these constituent compounds in ameliorating the diabetic complications [18,19,20,21,22,23].

Before commencement of an extraction process, the plant sample is required to undergo a pre-extraction treatment (drying and grinding) to increase the shelf-life, as well as to maximize the extraction of metabolites from the plant [24]. Drying method can be categorized into two types; thermal drying includes the application of heat on the plant sample (hot air-drying, oven-drying (OD), microwave-drying, and sun-drying), while non-thermal drying avoids direct heat to dry the sample (freeze-drying (FD) and air-drying (AD)) [25]. Herbal industries prefers to use oven and AD method in their commercial facilities, as these methods are cost effective and easy to accomplish [26]. The utilization of suitable solvent is one of the key aspects of extraction to give the highest metabolites recovery available in the plant sample. The use of high polarity solvents might affect the solubility of the non-polar compounds, likewise, upon the use of low polarity solvents [27]. The loss of metabolites that are not able to be extracted out may hinder the full capacity of a plant sample to be established as a good antihyperglycemic alternative regimen. Therefore, an efficient extraction process that distills and preserves the indispensable bioactive compounds, contributing to the hypoglycemic effect of a plant extract, is crucial.

Though the hypoglycemic bioactivity of this plant has been previously reported [14], the effects of the drying method and extracting solvent on *M. calabura* bioactive compounds and, hence, the hypoglycemic activity, has not been discovered. Therefore, this study aims to investigate the optimal combination of drying method and extracting solvents (ethanol:water) with appropriate ratio that can produce *M. calabura* extracts with the most desirable and potent in vitro anti-diabetic activities. In addition, an ultrahigh-performance-liquid chromatography-electrospray ionization tandem mass spectrometry (UHPLC-ESI-MS/MS) method was used to characterize the metabolites of the active extract, based on their MS/MS data, for the first time. The results obtained from this study will allow recommendations on the best combination of drying method and extracting solvent to obtain *M. calabura* extract with significant potential in selected bioactivity and high productivity.

## 2. Results and Discussion

### 2.1. Influence of Varied Drying Process and Ethanol Concentrations on Anti-Diabetic Activity

The bioactivity results of *M. calabura* leaves extracts, from different drying methods and ethanol:water ratios, are shown in Table 1. *M. calabura* leaves extracts were tested in two in vitro anti-diabetic assays, named α-glucosidase and α-amylase inhibition activity. In these two activities, the hypoglycemic potential of *M. calabura* extracts, to reduce the blood sugar level through competitive binding to the active site of the enzyme to prevent the conversion of carbohydrates to simple glucose, were determined [28]. The α-glucosidase inhibitory activity was designed to assess the ability of an extract to suppress the active enzyme from catalyzing the conversion of glucose from the disaccharides, which occurs in the small intestine [29]. In this study, PNPG, a specific substrate that allows hydrolyzation to 4-nitrophenol (yellow colored product) by α-glucosidase enzyme, quantitates at a maximum wavelength of 405 nm [30], while α-amylase inhibitory activity was used to measure the free carbonyl group of the reducing sugar (maltose) converted by the α-amylase from the complex carbohydrates (potato starch) [31]. The aldehyde group from the maltose reduces the yellow colored DNS to form 3-amino-5-nitrosalicylic acid (brick red colored solution) [31]. The concentration of maltose presence in the sample, determine the intensity of the color at a wavelength of 540 nm [32].

The results demonstrated in Table 1 show the IC_50_ value of the α-glucosidase inhibition activity of *M. calabura* leaves extracts, ranging from 0.46–2.76 µg/mL in comparison to quercetin (2.15 ± 0.26 µg/mL), while for α-amylase inhibition activity, the IC_50_ value, ranging from 23.84–185.17 µg/mL in comparison to acarbose (0.68 ± 0.14 µg/mL). In α-glucosidase inhibition activity, the effect of different ethanol:water ratios played the most significant role that affected α-glucosidase inhibition ability of *M. calabura* leaves compared to the different drying methods. The 50 and 100% ethanolic extracts from FD and AD leaves showed the lowest IC_50_ value with no significant difference (*p* > 0.05), ranging from 0.46 ± 0.05 to 1.07 ± 0.06 µg/mL. On the contrary, the IC_50_ value of 50% ethanolic extract from OD leaves was the lowest compared to that of the 100 and 0% ratios from the same drying method, whereas the 0% ethanolic extract from all the drying methods showed significantly lower α-glucosidase inhibition potential compared to that of other extracts. As for the α-amylase inhibition activity, the lowest IC_50_ value was found in the 50% ethanolic extract, regardless of its drying method, and in the 100% ethanolic extract from the FD method. When comparing the different drying methods, the FD *M. calabura* leaves showed the highest α-amylase inhibition capability compared to the AD and OD methods in both 50 and 100% ethanolic extracts. However, the OD leaves showed the lowest IC_50_ value when extracted with 0% ethanol. The use of 50% ethanol, evidently, was the best ethanol:water ratio to extract out the phytochemical constituents with hypoglycemic potential in *M. calabura* leaves for both tested bioactivities. In line with the previous in vitro study, where the use of alcohol and water mixture could efficiently extract out the wider spectrum of phytochemicals constituents that might contribute to the bioactivity as compared to a mono-component solvent system [33]. Besides that, the utilization of the 50% ethanolic ratio allows utilization of less organic solvent, increasing extract’s solubility in less toxic organic environment and confirming its usage harmlessly in in vivo studies [34].

In this study, FD and AD extracts were procured as the most effective drying methods in order to identify the α-glucosidase and α-amylase inhibitors followed by OD extraction. The absence of thermal degradation hindered the degradative enzyme to function [35]. Several studies have concurred that the FD method enables higher retention of phytochemical compounds that leads to enhanced biological activity of the plant [36]. In contrast, the thermal processing such as oven-drying, hot air-drying, and microwave-drying impeded the extraction of phytochemicals of the plant by thermal breakdown, disrupting the integrity of cell structure and migration of components, catalyzing further disruption by various chemical reactions such as enzymes, light, and oxygen [37]. In addition, the utilization of the FD method will shorten the extraction process by expediting the drying time, compared to the AD technique, as well as potentially providing higher yield of anti-diabetic phytochemical constituents [38,39]. Hence, the 50% ethanol:water ratio on FD *M. calabura* leaves, with strong α-glucosidase and α-amylase inhibitory activity, can be potentially exploited further to create a natural regimen from *M. calabura* leaves to aid in the blood sugar management of a diabetic patient.

### 2.2. UHPLC-ESI-MS/MS Characterization of Phytoconstituents in the M. calabura Leaves Extract

**Figure 1:**
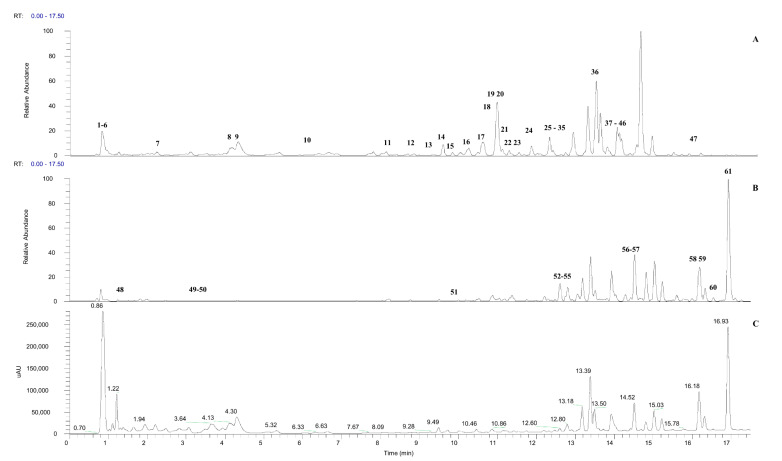
Total ion chromatogram (TIC) and UV-Vis chromatogram (254 nm) (**C**) of *M. calabura* FD leaves extracted with 50% ethanol in negative (**A**) and positive ion mode (**B**), respectively.

Based on the anti-diabetic activity of *M. calabura* leaves, the FD 50% ethanolic extract was identified and selected as the most active extract. This extract was then subjected to UHPLC-ESI-MS/MS identification to have better insight on the constituents present that might enhance the bioactivities. Figure 1 shows the total ion chromatogram and UV-Vis chromatogram (254 nm) of the FD 50% ethanolic *M. calabura* leaves extract for both positive and negative ion mode. A total of 61 compounds (Table 2) were putatively identified based on their molecular ions in the full scan mass spectra, retention time, fragmentation patterns, mass error, and UV absorption spectra supported with database mining, such as MassBank databases (https://massbank.eu/MassBank/), Human Metabolomics Databases (HMDB) (https://hmdb.ca/), Metabolomics Workbench databases (https://www.metabolomicsworkbench.org/), Chemspider databases (http://www.chemspider.com/), and MassFrontier (version 8.0) software (Thermo Scientific, Waltham, MA, USA). The compounds identified include five catechin derivatives, five types of kaempferol derivatives, three types of quercetin derivatives, one apigenin derivative compound, three types of luteolin derivatives, seven types of daidzein derivatives, 19 types of other flavonoids, two types of anthocyanin compounds, two types of chalcone compounds, six types of quinone derivatives, two types of lactones derivatives, two types of alkaloid derivatives, two types of sugar, one terpene glycoside, and one ellagitannins derivative.

#### 2.2.1. Catechin Derivatives

**Figure 2:**
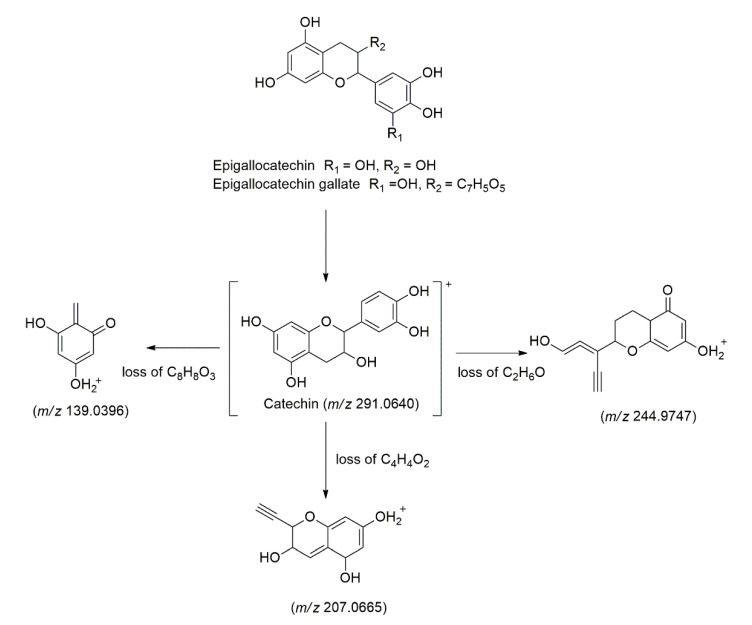
The main fragmentation pathways of the catechin derivatives.

Catechin (**Peak 48**) was identified at t_R_ = 1.68 min with protonated molecule [M+H]^+^ at *m/z* 291.0640. The fragmentation pattern in the MS/MS analysis for this compound were at *m/z* 244.9747 [M+H-46]^+^ (loss of C_2_H_6_O), 207.0665 [M+H-84]^+^ (loss of C_4_H_4_O_2_), and 139.0396 [M+H-152]^+^ (loss of C_8_H_8_O_3_) [40]. Epigallocatechin (**Peak 3**) and gallocatechin (**Peak 6**) were identified at t_R_ = 1.24 and 1.40 min, respectively, with a deprotonated molecule [M-H]^−^ at *m/z* 305.0668. The ions later produced three fragmentation ions in the MS/MS analysis at *m/z* 289.0233 as the base peak of catechin, 245.0284 [M-H-60]^−^ due to loss of C_2_H_4_O_2_, 219.0655 [M-H-86]^−^ due to loss of C_3_H_2_O_3_ and 167.0338 [M-H-138]^−^ due to the loss of C_7_H_6_O_3_ [40]. Figure 2 demonstrates the common fragmentation pathways of the catechin derivatives. Another catechin derivatives named epigallocatechin gallate (**Peak 51**) was identified at t_R_ = 9.68 min with protonated molecule [M+H]^+^ at *m/z* 459.0942, with three fragmentation ions in the MS/MS analysis at *m/z* 321.0591 [M+H-138]^+^ due to dissociation of C_7_H_6_O_3_, 289.0771 [M+H-170]^+^ as the base peak of catechin, and 275.0818 [M+H-184]^+^ due to loss of C_8_H_8_O_5_ [40].

#### 2.2.2. Kaempferol Derivatives

**Figure 3:**
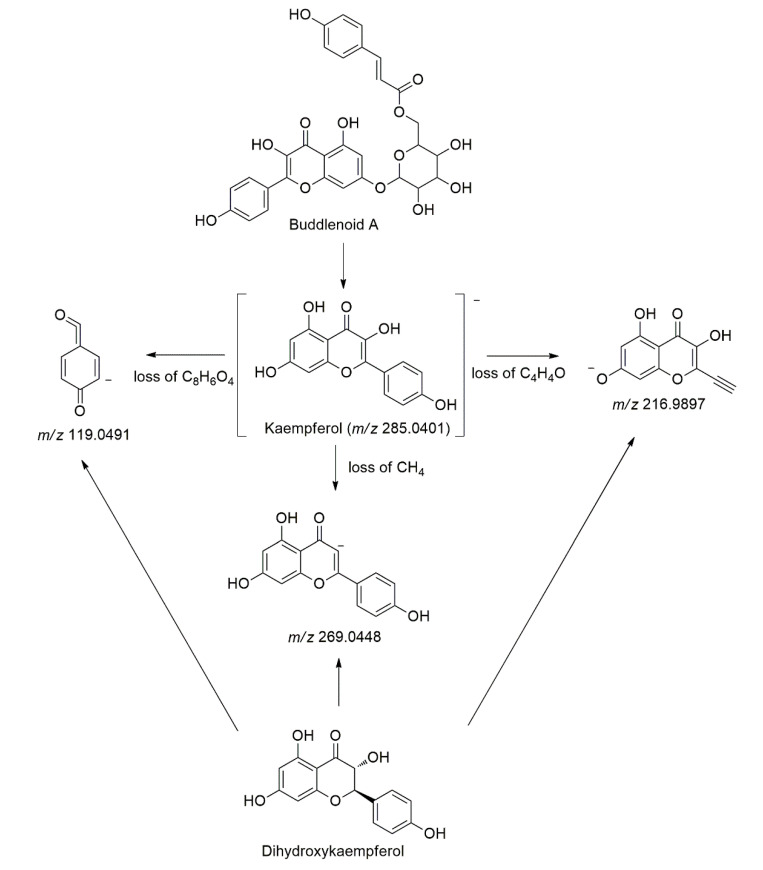
The main fragmentation pathways of the kaempferol derivatives.

Kaempferol (**Peak 24**) was identified at t_R_ = 11.87 min with a deprotonated molecule [M-H]^−^ at *m/z* 285.0401, which later produced three characteristic fragment ions in the MS/MS analysis at *m/z* 269.0448 [M-H-16]^−^ due to the loss of CH_4_, *m/z* 216.9897 [M-H-68]^−^ due to the loss of C_4_H_4_O and *m/z* 119.0491 [M-H-166]^−^ due to the loss of C_8_H_6_O_4_ [40]. Identification of kaempferol was confirmed with the authentic standard. Buddlenoid A (**Peak 12**) and its isomer (**Peak 14**) were identified at (t_R_ = 9.43 and 9.75 min). These compounds showed a deprotonated molecule [M-H]^−^ at *m/z* 593.1304, which later produced six characteristic fragment ions in the MS/MS analysis at *m/z* 447.0935 [M-H-146]^−^, 429.0823 [M-H-164]^−^, and 285.0404 [M-H-308]^−^. The fragmentations occur due to the loss of C_9_H_6_O_2_, C_9_H_8_O_3_, and C_15_H_16_O_7_, respectively. Besides that, the compounds also show a fragmentation pattern typical of kaempferol aglycone at *m/z* 269.0448, 216.9897, and 119.0491 [41]. This compound was previously isolated and characterized in this plant by Nshimo et al. [42]. Figure 3 depicts the main fragmentation pathways of the kaempferol derivatives. Buddlenoid A dimer (**Peak 13**) was also identified in abundance at t_R_ = 9.49 min with a deprotonated molecule [M-H]^−^ at *m/z* 1187.2678. This compound exhibited the same fragmentation patterns in the MS/MS analysis as buddlenoid A, with double of its monomer [41]. Another kaempferol derivative was also identified in the chromatogram at t_R_ = 13.63 min, with a deprotonated molecule [M-H]^−^ at *m/z* 287.0539. Additionally, *m/z* 271.0607 [M-H-16]^−^ occurs due to loss of CH_4_ in the MS/MS analysis, while *m/z* 269.0723, 216.9894, and 119.0077 are typical fragments for kaempferol derivatives [40]. Hence, this compound was identified as dihydrokaempferol (**Peak 37**).

#### 2.2.3. Quercetin Derivatives

**Figure 4:**
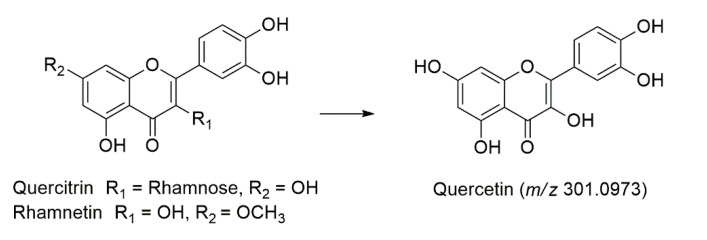
The main fragmentation pathways of the quercetin derivatives.

Quercitrin (**Peak 21**) and its isomer (**Peak 23**) was identified at t_R_ = 11.02 and 11.17 min, with a deprotonated molecule [M-H]^−^ at *m/z* 447.2237, which later produced three characteristic fragment ions in the MS/MS analysis at *m/z* 301.1495 [M-H-146]^−^ as the base peak for quercetin, *m/z* 285.2063 [M-H-162]^−^ due to the loss of C_6_H_10_O_5_ and *m/z* 245.0717 [M-H-202]^−^ due to the loss of C_8_H_10_O_6_ [40]. Identification of this compound was further confirmed with the authentic standard. Rhamnetin (**Peak 52**) were also characterized at t_R_ = 12.58 min based on its protonated molecule [M+H]^+^ at *m/z* 317.0671 and the three fragmentation ions in the MS/MS analysis. At *m/z* 301.0973 [M+H-16]^+^ arose as the base peak for quercetin, as shown in Figure 4. The *m/z* 285.0734 [M+H-31]^+^ and *m/z* 245.1063 [M+H-71]^+^ occurred due to the loss of CH_3_O and C_3_H_3_O_2_, respectively [40].

#### 2.2.4. Apigenin Derivatives

**Figure 5:**
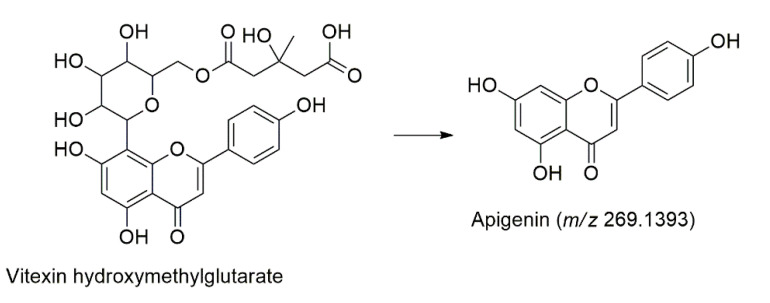
The fragmentation pathways of the apigenin derivatives.

One apigenin derivatives named vitexin hydroxymethylglutarate (**Peak 30**) was identified at t_R_ = 12.72 min, based on its deprotonated molecule [M-H]^−^ at *m/z* 571.748 and the three fragmentation ions in the MS/MS analysis. The product ions were *m/z* 513.0697 [M-H-62]^−^ due to CH_2_O_3_, *m/z* 341.1581 [M-H-234]^−^ occur due to loss of C_9_H_14_O_7_ and *m/z* 269.1393 [M-H-306]^−^ as the base peak of apigenin, as shown in Figure 5 [43].

#### 2.2.5. Luteolin Derivatives

**Figure 6:**
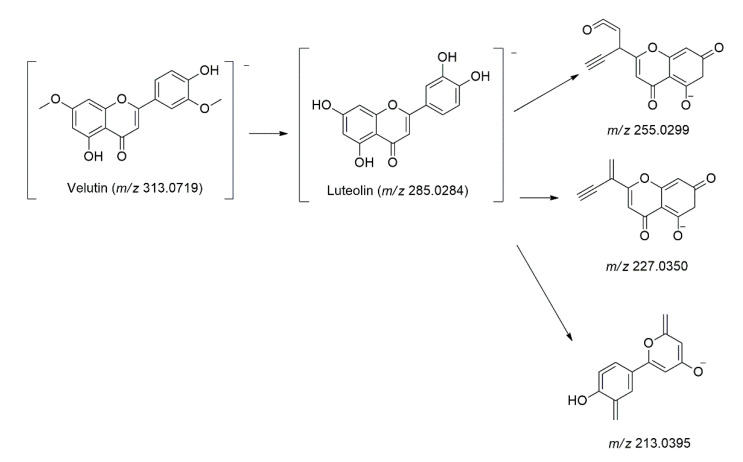
The fragmentation pathways of the luteolin derivatives.

One luteolin derivative named velutin (**Peak 40**) was tentatively identified, as they share three significant product ions [M-H]^−^ at *m/z* 285.0284, 255.0299, 227.0350, and 213.0395, which were the base peaks for luteolin aglycone [40]. Velutin and its isomers (**Peak 40, 45, and 46**) were identified based on its deprotonated molecule [M-H]^−^ at *m/z* 313.0719 at t_R_ = 14.03, 15.01, and 15.19 min [40]. Figure 6 shows the main fragmentation pathways found in luteolin derivatives.

#### 2.2.6. Daidzein Derivatives

**Figure 7:**
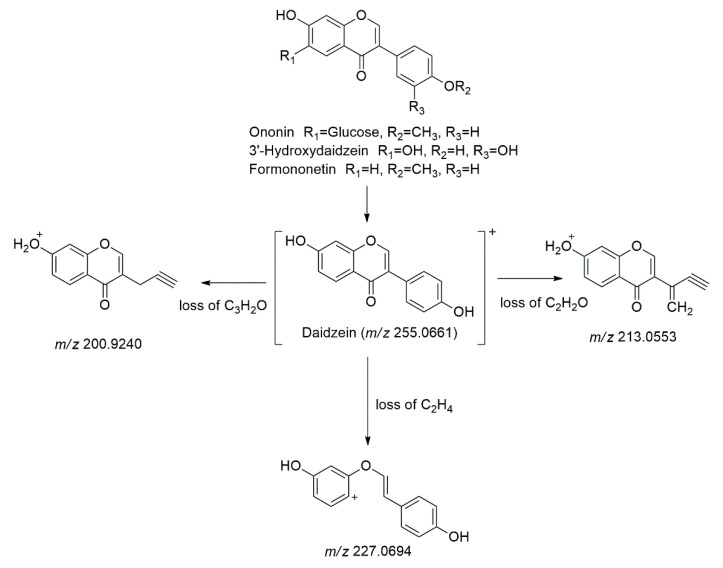
The fragmentation pathways of the daidzein derivatives.

Daidzein (**Peak 54**) and its isomer (**Peak 55**) were identified with a protonated molecule [M+H]^+^ at *m/z* 255.0661 at t_R_ = 13.13 and 13.29 min, respectively. This compound was further characterized with the three fragmentation ions in MS/MS MS/MS analysis at *m/z* 227.0694 [M+H-28]^+^ with the loss of C_2_H_4_, *m/z* 213.0553 [M+H-42]^+^, with loss of C_2_H_2_O and *m/z* 200.9240 [M+H-54]^+^, and with the loss of C_3_H_2_O [40]. Identification of daidzein was confirmed with the authentic standard. Three of daidzein derivatives were also identified in the sample named ononin (**Peak 53**), 3′-hydroxydaidzein (**Peak 56**), and formononetin (**Peak 58**). Ononin (**Peak 53**) was identified at t_R_ = 13.00 min based on its protonated molecule [M+H]^+^ at *m/z* 431.1330 and the fragmentation ions at MS/MS analysis that are similar to the base peak of daidzein, as shown in Figure 7 [40]. Additionally, 3′-hydroxydaidzein (**Peak 56**) and its isomer (**Peak 57**) with protonated molecule [M+H]^+^ at *m/z* 271.0611 were tentatively identified based on their similar base peak, as of daidzein, as demonstrated in Figure 7 at t_R_ = 14.38 and 14.78 min, respectively [44]. Then, 3′-hydroxydaidzein was previously isolated and characterized in this plant by Matsuda et al. [45]. While at t_R_ = 16.13 and 16.29, formononetin (**Peak 58**) and its isomer (**Peak 59**) were identified based on its protonated molecule [M+H]^+^ at *m/z* 269.0819 and the four fragmentation ions at MS/MS analysis that agreed to the base peak of daidzein [40]. The identification of this compound was confirmed with the authentic standard.

#### 2.2.7. Other Flavonoids

Additionally, 3′-Hydroxy-7,8,4′,5′-tetramethoxyflavone (**Peak 4**) was identified at t_R_ = 1.36 min with a deprotonated molecule [M-H]^−^ at *m/z* 357.1195, which later produced three fragmentation ions at MS/MS analysis at *m/z* 270.8385 [M-H-86]^−^ due to the cleavage of C_4_H_6_O_2_, *m/z* 224.8610 [M-H-132]^−^ due to the cleavage of C_6_H_12_O_3_, and *m/z* 179.0586 [M-H-178]^−^ due to cleavage of C_10_H_10_O_3_ [46]. This compound was previously isolated and characterized in this plant by Kaneda et al. [46].

**Peak 7** was identified as 7,8,3′,4′,5′-pentamethoxyflavone at t_R_ = 2.42 min with a deprotonated molecule [M-H]^−^ at *m/z* 371.0986. This ion was then further fragmented in MS/MS analysis and produced three fragmentation ions at *m/z* 296.98684 [M-H-74]^−^ with the loss of C_3_H_6_O_2_, *m/z* 240.8795 [M-H-130]^−^ with the loss of C_6_H_10_O_3_, and *m/z* 231.0506 [M-H-140]^−^ with the loss of C_7_H_8_O_3_ [46]. Previously, 7,8,3′,4′,5′-pentamethoxyflavone was also isolated and characterized in *M. calabura* by Kaneda et al. [46].

At t_R_ = 8.70 min, another flavonoid named hiravanone (**Peak 11**) was identified based on its deprotonated molecule [M-H]^−^ at *m/z* 437.0466, with three fragmentation ions in MS/MS analysis at *m/z* 296.8747 [M-H-140]^−^, 288.7813 [M-H-148]^−^, and 242.8798 [M-H-194]^−^ due to the loss of C_7_H_8_O_3_, C_9_H_8_O_2_, and C_10_H_10_O_4_, respectively [47]. This compound was previously isolated and characterized in this plant by Seo et al. [47].

Then, 3,5,8-Trihydroxy-7-methoxyflavanone (**Peak 16**) and its isomers (**Peak 17 and 18**) were identified based on its deprotonated molecule [M-H]^−^ at *m/z* 301.0721 at t_R_ = 10.47, 10.63, and 10.78 min, respectively, with three fragmentation ions in the MS/MS analysis at *m/z* 286.0490 [M-H-14]^−^ due to the loss of CH_2_, *m/z* 269.0465, due to the loss of CH_4_O, and *m/z* 211.0475 [M-H-90]^−^ due to the loss of C_7_H_6_ [16]. Previously, 3,5,8-Trihydroxy-7-methoxyflavanone was isolated and characterized in the leaves of *M. calabura* by Su et al. [16].

A deprotonated molecule [M-H]^−^ at *m/z* 237.0555 and its two fragmentation ions in MS/MS analysis at *m/z* 209 [M-H-28]^−^ occur due to loss of CO, and *m/z* 160 [M-H-77]^−^ occurs due to the loss of C_6_H_5_, which was identified as hydroxyflavone (**Peak 22**) at t_R_ = 11.13 min [40].

At t_R_ = 12.30 min, 6,8-diprenyleriodictyol (**Peak 25**) was tentatively identified with the deprotonated molecule [M-H]^−^ at *m/z* 423.0928. Three fragmentation ions occur in MS/MS analysis due to loss of C_4_H_6_O, C_8_H_7_O_2_, and C_11_H_8_O_2_ at *m/z* 353.2442 [M-H-70]^−^, 287.6386 [M-H-135]^−^, and 251.0923 [M-H-172]^−^, respectively [40]. This compound was previously isolated and characterized in this plant by Seo et al. [47].

Cirsiliol (**Peak 27**) and its isomer (**Peak 28**) were putatively identified at t_R_ = 12.50 and 12.66 min, based on its deprotonated molecule [M-H]^−^ at *m/z* 329.0672 and its fragmentation ions in MS/MS analysis at *m/z* 314.0419 [M-H-15]^−^, due to the loss of CH_3_, *m/z* 299.0198 [M-H-30]^−^ due to the loss of CH_2_O and *m/z* 285.0399 [M-H-44]^−^ due to the loss of C_2_H_4_O [40].

At deprotonated molecule [M-H]^−^ *m/z* 343.0823 with t_R_ = 12.96 min, a compound named 8,3′-dihydroxy-7,4′,5′-trimethoxyflavone (**Peak 32**) was identified with three characteristic fragmentation ions in MS/MS analysis at *m/z* 327.0524 [M-H-16]^−^, 313.0357 [M-H-30]^−^, and 256.9828 [M-H-86]^−^. These ions produced, due to the loss of CH_4_, CH_2_O and C_4_H_6_O_2_, respectively [46]. Previously, 8,3′-dihydroxy-7,4′,5′-trimethoxyflavone was isolated and characterized in this plant species by Kaneda et al. [46].

Dimethoxyhydroxyflavanone (**Peak 33**) and its isomer (**Peak 36**) were identified based on its deprotonated molecule [M-H]^−^ at *m/z* 299.0196 at t_R_ = 13.31 and 13.47 min. This ion later produced four fragmentation ions in MS/MS analysis at *m/z* 284.0326 [M-H-15]^−^ due to the loss of CH_3_, *m/z* 269.0457 [M-H-30]^−^ due to the loss of CH_2_O, *m/z* 255.0300 [M-H-44]^−^ due to the loss of CO_2_, and *m/z* 239.0348 [M-H-60]^−^ due to the loss of C_3_H_8_O [40].

Kievitone (**Peak 38**) was identified based on its deprotonated molecule [M-H]^−^ at *m/z* 355.1038 at t_R_ = 13.67 min, which later produced three fragmentation ions in MS/MS analysis at *m/z* 285.0984 [M-H-70]^−^ with the loss of C_3_H_2_O_2_, *m/z* 255.0656 [M-H-100]^−^ with the loss of C_4_H_4_O_3_, and *m/z* 241.1012 [M-H-114]^−^ with the loss of C_6_H_10_O_2_ [48].

Additionally, 6-Hydroxyflavanone (**Peak 43**) was identified at t_R_ = 14.61 min. This compound gives out a deprotonated molecule [M-H]^−^ at *m/z* 239.0713 and three fragmentation ions in MS/MS analysis at *m/z* 211.0628 [M-H-28]^−^, 197.0602 [M-H-42]^−^, and 136.0112 [M-H-103]^−^. These fragmentations occurred due to the loss of CO, C_2_H_2_O, and C_8_H_7_, respectively [40]. The presence of this compound was confirmed with the available standards.

At t_R_ = 14.98 min, with a deprotonated molecule [M-H]^−^ at *m/z* 393.3017, which later produced three fragmentation ions in MS/MS analysis at *m/z* 375.2910 [M-H-18]^−^, *m/z* 361.2715 [M-H-32]^−^ and *m/z* 353.2982 [M-H-40]^−^ was identified as trihydroxydiprenylisoflavan (**Peak 44**), as this fragmentation occurred due to the loss of H_2_O, CH_4_O, and C_3_H_4_, respectively [49].

Next, 3-hydroxy-3′,4′-dimethoxyflavone (**Peak 60**) and its isomer (**Peak 61**) were identified putatively at t_R_ = 16.86 and 17.02 min. This compound give out to a deprotonated molecule [M+H]^+^ at *m/z* 299.0925, which later produced three significant fragmentation ions in MS/MS analysis at *m/z* 283.0610 [M+H-16]^+^, 267.0578 [M+H-32]^+^, and 237.0548 [M+H-62]^+^. Each of which occurred due to the loss of one oxygen moiety, loss of CH_4_O, and loss of C_2_H_6_O_2_, respectively [40]. Additionally, 3-hydroxy-3′,4′-dimethoxyflavone was isolated and elucidated from the leaves of *M. calabura* by Sufian et al. [12].

At t_R_ = 3.66 and 3.82 min, myricitrin (**Peak 49**) and its isomer (**Peak 50**) were identified with deprotonated molecule [M+H]^+^ at *m/z* 465.1050, produced three significant fragmentation ions in MS/MS analysis at *m/z* 447.3480 [M+H-18]^+^, 303.0513 [M+H-162]^+^, and 285.0772 [M+H-180]^+^. These fragmentations occur due to the loss of H_2_O, C_6_H_10_O_5_, and C_6_H_12_O_6_, respectively [40].

#### 2.2.8. Anthocyanin

Myrtillin (**Peak 8**) and its isomer (**Peak 9**) were identified based on its deprotonated molecule [M-H]^−^ at *m/z* 464.8049 at t_R_ = 4.05 and 4.21 min, which later produced three fragmentation ions in MS/MS analysis at *m/z* 286.9921 [M-H-78]^−^, 299.0193 [M-H-166]^−^, and 178.9979 [M-H-286]^−^. These fragmentations occurred due to the loss of C_3_H_10_O_2_, C_6_H_14_O_5_, and C_15_H_10_O_6_, respectively [40]. This compound was isolated and characterized by Harborne et al. [50] in this plant.

#### 2.2.9. Chalcone

At t_R_ = 10.16 and 12.73 min, chalcone compounds were identified based on its deprotonated molecule [M-H]^−^ at *m/z* 255.0662 and the similar fragmentation ions in MS/MS analysis at *m/z* 227.0477 [M-H-28]^−^ due to the loss of C_2_H_4_, *m/z* 213.0526 [M-H-42]^−^ due to the loss of C_2_H_2_O, and *m/z* 187.0655 [M-H-68]^−^ due to the loss of C_4_H_4_O. These compounds were identified as isoliquiritigenin (**Peak 15**) and its isomers (**Peak 31**) [51]. Isoliquiritigenin was previously isolated and characterized by Hsu et al. [51] in *M. calabura* species.

#### 2.2.10. Quinone Derivatives

Six different quinone derivatives and their isomers, named arbutin, rhein, and lapachol, were tentatively identified throughout the spectrum. At t_R_ = 10.80 and 10.96 min, arbutin (**Peak 19**) and its isomer (**Peak 20**) with a deprotonated molecule [M-H]^−^ at *m/z* 271.0613 and three fragmentation ions in MS/MS analysis at *m/z* 253.0507 [M-H-18]^−^, 197.0603 [M-H-74]^−^, and 161.0600 [M-H-110]^−^ due to the loss of H_2_O, C_3_H_6_O_2_, and C_6_H_6_O_2_, respectively [52]. Rhein (**Peak 35**) and its isomers were tentatively identified at t_R_ = 13.45, 13.92, and 14.23 min, with its deprotonated molecule at [M-H]^−^ at *m/z* 282.9547 and three fragmentation ions in MS/MS analysis at *m/z* 267.0296 [M-H-16]^−^ with the loss of CH_4_, 239.0353 [M-H-48]^−^ with the loss of CH_4_O_2_, and 211.0396 [M-H-72]^−^ with the loss of C_3_H_4_O_2_ [53]. While lapachol (**Peak 42**) was identified at t_R_ = 14.38 min, with its deprotonated molecule at [M-H]^−^ at *m/z* 240.9106, and three fragmentation ions at MS/MS analysis at *m/z* 223.0756 [M-H-18]^−^, 198.1011 [M-H-43]^−^, and 186.0566 [M-H-55]^−^ due to the loss of H_2_O, C_2_H_3_O, and C_4_H_7_, respectively [54].

#### 2.2.11. Lactone Derivatives

One lactone derivative named yangonin (**Peak 29**) and its isomer (**Peak 34**) were identified at t_R_ = 12.71 and 13.40 min with deprotonated molecule at [M-H]^−^ at *m/z* 257.0644. Three similar fragmentation ions in MS/MS analysis includes *m/z* 239.0710 [M-H-18]^−^ (loss of H_2_O), 213.0919 [M-H-44]^−^ (loss of CO_2_), and 197.0815 [M-H-60]^−^ (loss of C_2_H_4_O_2_) [50].

#### 2.2.12. Terpene Glycoside

Geniposide (**Peak 10**) with a deprotonated molecule at [M-H]^−^ at *m/z* 387.1145 was identified at t_R_ = 6.34 min, with three MS/MS fragmentation ions at *m/z* 284.0327 [M-H-103]^−^, 255.0298 [M-H-132]^−^, and 224.8609 [M-H-162]^−^. These signals, respectively, corresponded to the loss of C_4_H_7_O_3_, C_5_H_8_O_4_, and C_6_H_10_O_5_ [55]. Identification of this compound was confirmed by using the authentic standard.

#### 2.2.13. Alkaloid Derivatives

Two alkaloid derivatives identified were piceatannol galloylglucoside (**Peak 26**) with deprotonated molecule at [M-H]^−^ at *m/z* 557.1458 and narceinone (**Peak 47**) with deprotonated molecule at [M-H]^−^ at *m/z* 457.9947 at t_R_ = 12.36 and 16.18 min, respectively. Piceatannol galloylglucoside (**Peak 26**) give out to four fragmentations ions in MS/MS analysis at *m/z* 301.1331 [M-H-256]^−^ (loss of C_11_H_12_O_7_), 285.0776 [M-H-272]^−^ (loss of C_11_H_12_O_8_), 257.3555 [M-H-300]^−^ (loss of C_16_H_12_O_6_), and 201.0187 [M-H-356]^−^ (loss of C_15_H_16_O_10_), while narceinone (**Peak 47**) give out to three fragmentations ions in MS/MS analysis at *m/z* 427.9972 [M-H-30]^−^, 397.9993 [M-H-59]^−^, and 367.9799 [M-H-90]^−^ due to the loss of CH_2_O, C_2_H_3_O_2_, and C_2_H_2_O_4_, respectively [56,57].

#### 2.2.14. Sugar

Sucrose (**Peak 2**) and its dimer (**Peak 1**) were identified at t_R_ = 0.94 and 0.81 min, respectively with deprotonated molecule at [M-H]^−^ at *m/z* 341.1090 and 683.2253. They share a similar fragmentations pattern in MS/MS analysis at *m/z* 179.0555 [M-H-162]^−^ (loss of C_6_H_10_O_5_), 161.0443 [M-H-180]^−^ (loss of glucose moiety), and 143.0345 [M-H-198]^−^ (loss of C_6_H_14_O_7_) [40].

#### 2.2.15. Ellagitannin Derivative

Corilagin (**Peak 5**) was tentatively identified at t_R_ = 1.39 min with deprotonated molecule at [M-H]^−^ at *m/z* 633.0735. The three fragmentations pattern in MS/MS analysis were shown at *m/z* 481.1787 [M-H-152]^−^ (loss of C_7_H_4_O_4_), 313.0568 [M-H-320]^−^ (loss of C_14_H_8_O_9_), and 169.0137 [M-H-164]^−^ (loss of C_20_H_16_O_13_) [58].

**Table 2:** Tentative identification of compounds present in *M. calabura* FD leaves extracted with 50% ethanol.

Peak No	t_R_	UV λ-Max	MF	Exact Mass	(M-H)^−^	(M+H)^+^	Mass Error	MS/MS Fragment Ions	Tentative Identification	References
1	0.81	210, 274, 350, 370	ND	ND	683.2253	−	ND	341.1091, 179.0554, 161.0447, 143.0340	Sucrose dimer	[40]
2	0.94	210, 272, 350, 370	C_12_H_22_O_11_	342.1162	341.1090	−	0.0072	179.0555, 161.0443, 143.0345	Sucrose	[40]
3	1.24	208, 266, 292, 350, 370	C_15_H_14_O_7_	306.0740	305.0668	−	0.0072	289.0642, 245.0440, 219.0657, 167.0341	Epigallocatechin	[40]
4	1.36	210, 272	C_19_H_18_O_7_	358.1053	357.1195	−	−0.0142	270.8385, 224.8610, 179.0586	3′-Hydroxy-7,8,4′,5′-tetramethoxyflavone *	[46]
5	1.39	208, 216, 276, 270	C_27_H_22_O_18_	634.0806	633.0735	−	0.0071	481.1787, 313.0568, 169.0137	Corilagin	[58]
6	1.40	208, 274	C_15_H_14_O_7_	306.0740	305.0668	−	0.0072	289.0233, 245.0284, 219.0655, 167.0338	Gallocatechin	[40]
7	2.42	208, 356	C_20_H_20_O_7_	372.1209	371.0986	−	0.0223	296.8684, 240.8795, 231.0506	7,8,3′,4′,5′-Pentamethoxyflavone *	[46]
8	4.05	206, 256, 350	C_21_H_21_O_12_	465.1033	464.8049	−	−0.7016	386.9921, 299.0193, 178.9979	Myrtillin *	[40]
9	4.21	206, 258, 350	C_21_H_21_O_12_	465.1033	464.8049	−	−0.7016	386.9907, 298.9871, 178.9980	Myrtillin isomer	[40]
10	6.34	210, 272, 350, 370	C_17_H_24_O_10_	388.1369	387.1145	−	0.0224	284.0327, 255.0298, 224.8609	Geniposide #	[40]
11	8.70	222, 272	C_26_H_30_O_6_	438.2042	437.0466	−	0.1576	296.8747, 288.7813, 242.8798	Hiravanone *	[47]
12	9.43	222, 274	C_30_H_26_O_13_	594.1373	593.1304	−	0.0069	447.0935, 429.0823, 285.0404	Buddlenoid A *	[41]
13	9.49	222, 268, 314	ND	ND	1187.2678	−	ND	593.1306, 447.0932, 429.0825, 285.0408	Buddlenoid A dimer	[41]
14	9.75	222, 268, 296, 374	C_30_H_26_O_13_	594.1373	593.1304	−	0.0069	447.0919, 429.0820, 285.0406	Buddlenoid A isomer	[41]
15	10.16	222, 280	C_15_H_12_O_4_	256.0736	255.0662	−	0.0074	227.0477, 213.0526, 187.0655	Isoliquiritigenin *	[51]
16	10.47	222, 294	C_16_H_14_O_6_	302.0790	301.0721	−	0.0069	286.0490, 269.0465, 211.0475	3,5,8-Trihydroxy-7-methoxyflavanone *	[16]
17	10.63	224, 286	C_16_H_14_O_6_	302.0790	301.0721	−	0.0069	286.0489, 269.0461, 211.0474	Trihydroxymethoxyflavanone isomer	[16]
18	10.78	222, 294	C_16_H_14_O_6_	302.0790	301.0721	−	0.0069	286.0492, 269.0452, 211.0475	Trihydroxymethoxyflavanone isomer	[16]
19	10.80	222, 294	C_12_H_16_O_7_	272.0896	271.0613	−	0.0283	253.0507, 197.0603, 161.0600	Arbutin	[52]
20	10.96	224, 276, 296, 376	C_12_H_16_O_7_	272.0896	271.0613	−	0.0283	253.0509, 197.0603, 161.0600	Arbutin isomer	[52]
21	11.02	224, 282	C_21_H_20_O_11_	448.1006	447.2237	−	−0.1231	301.1495, 285.2063, 245.0717	Quercitrin #	[40]
22	11.13	224, 290, 298	C_15_H_10_O_3_	238.0630	237.0555	−	0.0075	209.0591, 160.0157	Hydroxyflavone	[40]
23	11.17	224, 306	C_21_H_20_O_11_	448.1006	447.2237	−	−0.1231	301.0923, 285.0311, 245.0662	Quercitrin isomer	[40]
24	11.87	224, 282	C_15_H_10_O_6_	286.0477	285.0401	−	0.0076	269.0448, 216.9897, 119.0491	Kaempferol #	[40]
25	12.30	224, 270, 292, 312	C_25_H_28_O_6_	424.1886	423.0928	−	0.0958	353.2442, 287.6386, 251.0923	6,8-Diprenyleriodictyol *	[47]
26	12.36	224, 290	C_27_H_26_O_13_	558.4875	557.1458	−	0.3417	301.1331, 285.0776, 257.3555, 201.0187	Piceatannol galloylglucoside	[57]
27	12.50	224, 286	C_17_H_14_O_7_	330.0740	329.0672	−	0.0068	314.0419, 299.0198, 285.0399	Cirsiliol	[40]
28	12.66	224, 292, 376	C_17_H_14_O_7_	330.0740	329.0670	−	0.0070	314.0435, 299.0198, 285.0421	Cirsiliol isomer	[40]
29	12.71	224, 292, 376	C_15_H_14_O_4_	258.0892	257.0644	−	0.0248	239.0710, 213.0919, 197.0815	Yangonin	[50]
30	12.72	224, 292, 376	C_27_H_28_O_14_	576.1479	575.1748	−	−0.0269	513.0697, 341.1581, 269.1393, 231.1236	Vitexin hydroxymethylglutarate	[43]
31	12.73	224, 282, 332, 374	C_15_H_12_O_4_	256.0736	255.0662	−	0.0074	227.0711, 213.0549, 187.0634	Isoliquiritigenin isomer	[51]
32	12.96	224, 332, 374	C_18_H_16_O_7_	344.0896	343.0823	−	0.0073	327.0524, 313.0357, 256.9828	8,3′-Dihydroxy-7,4′,5′-trimethoxyflavone *	[46]
33	13.31	224, 280, 378	C_17_H_16_O_5_	300.0998	299.0196	−	0.0802	284.0326, 269.0457, 255.0300, 239.0348	Dimethoxyhydroxyflavanone	[40]
34	13.40	218, 274, 366	C_15_H_14_O_4_	258.0892	257.0644	−	0.0248	239.0712, 213.0915, 197.0597	Yangonin isomer	[50]
35	13.45	224, 272, 360, 374	C_15_H_8_O_6_	284.0321	282.9547	−	0.0774	267.0296, 239.0353, 211.0396	Rhein	[53]
36	13.47	222, 268, 312, 360	C_17_H_16_O_5_	300.0998	299.0196	−	0.0802	284.0326, 269.0816, 255.0304, 239.0346	Dimethoxyhydroxyflavanone isomer	[40]
37	13.63	224, 278, 356, 376	C_15_H_12_O_6_	288.0634	287.0539	−	0.0095	271.0607, 269.0723, 216.9894, 119.0077	Dihydrokaempferol	[40]
38	13.67	224, 286, 376	C_20_H_20_O_6_	356.1260	355.1038	−	0.0222	285.0948, 255.0656, 241.1012	Kievitone	[48]
39	13.92	224, 274, 352	C_15_H_8_O_6_	284.0321	282.9547	−	0.0774	267.0282, 239.0348, 211.0397	Rhein isomer	[53]
40	14.03	224, 268, 346	C_17_H_14_O_6_	314.0790	313.0719	−	0.0071	299.0505, 285.0284, 255.0299, 227.0350, 213.0395	Velutin	[40]
41	14.23	224, 378	C_15_H_8_O_6_	284.0321	282.9547	−	0.0774	267.0306, 239.0348, 211.0396	Rhein isomer	[53]
42	14.38	224, 286, 362, 376	C_15_H_14_O_3_	242.0943	240.9106	−	0.1837	223.0756, 198.1011, 186.0566	Lapachol	[54]
43	14.61	224, 376	C_15_H_12_O_3_	240.0786	239.0713	−	0.0073	211.0628, 197.0602, 136.0112	6-Hydroxyflavanone #	[40]
44	14.98	226, 274, 312, 378	C_25_H_30_O_4_	394.5033	393.3017	−	0.2016	375.2910, 361.2715, 353.2982	Trihydroxydiprenylisoflavan	[49]
45	15.01	220, 274, 316	C_17_H_14_O_6_	314.0790	313.0719	−	0.0071	299.0490, 285.0284, 255.0299, 227.0336, 213.0398	Velutin isomer	[40]
46	15.19	224, 270, 326, 374	C_17_H_14_O_6_	314.0790	313.0719	−	0.0071	299.0466, 285.0287, 255.0299, 227.0347, 213.0396	Velutin isomer	[40]
47	16.18	216, 270	C_23_H_25_NO_9_	459.4459	457.9947	−	0.4512	427.9972, 397.9993, 367.9799	Narceinone	[56]
48	1.68	208, 274	C_15_H_14_O_6_	290.0790	−	291.0640	0.0150	244.9747, 207.0665, 139.0396	Catechin	[40]
49	3.66	208, 254, 356, 376	C_21_H_20_O_12_	464.0955	−	465.1050	−0.0095	447.3480, 303.0513, 285.0772	Myricitrin	[40]
50	3.82	208, 254, 356, 376	C_21_H_20_O_12_	464.0955	−	465.1050	−0.0095	447.3459, 303.0511, 285.0771	Myricitrin isomer	[40]
51	9.68	222, 296, 374, 386	C_22_H_18_O_11_	458.0849	−	459.0942	−0.0093	321.0591, 289.0771, 275.0818	Epigallocatechin gallate	[40]
52	12.58	222, 294, 344, 378	C_16_H_12_O_7_	316.0583	−	317.0671	−0.0088	301.0973, 285.0734, 245.1063	Rhamnetin	[40]
53	13.00	226, 292, 326	C_22_H_22_O_9_	430.1260	−	431.1330	−0.0070	269.1334, 254.0331, 227.0704, 213.0568, 201.0357	Ononin	[40]
54	13.13	224, 274, 376	C_15_H_10_O_4_	254.0579	−	255.0661	−0.0082	227.0694, 213.0553, 200.9240	Daidzein #	[40]
55	13.29	224, 280, 378	C_15_H_10_O_4_	254.0579	−	255.0661	−0.0082	227.0668, 213.0560, 200.9234	Daidzein isomer	[40]
56	14.38	224, 282, 356, 378	C_15_H_10_O_5_	270.0528	−	271.0611	−0.0083	254.0868, 227.0920, 213.0558, 201.0650	3′-Hydroxydaidzein *	[44]
57	14.78	224, 338	C_15_H_10_O_5_	270.0528	−	271.0611	−0.0083	254.0666, 227.0918, 213.0557, 201.0810	Hydroxydaidzein isomer	[44]
58	16.13	226, 272, 378	C_16_H_12_O_4_	268.0736	−	269.0819	−0.0083	254.0582, 227.0666, 213.0548, 201.0915	Formononetin #	[40]
59	16.29	226, 266, 290, 354, 376	C_16_H_12_O_4_	268.0736	−	269.0819	−0.0083	254.0583, 227.0669, 213.0546, 201.0601	Formononetin isomer	[40]
60	16.86	226, 270	C_17_H_14_O_5_	298.0841	−	299.0925	−0.0084	283.0610, 267.0578, 237.0548	3-Hydroxy-3′,4′-dimethoxyflavone *	[40]
61	17.02	226	C_17_H_14_O_5_	298.0841	−	299.0925	−0.0084	283.0611, 267.0571, 237.0637	Hydroxydimethoxyflavone isomer	[40]

t_R_ = retention time; MF = molecular formula; * Indicate the compounds previously isolated from *M. calabura*; # indicated the compounds that were confirmed with the authentic standard.

### 2.3. UHPLC Absolute Quantification

Six standard compounds named geniposide, daidzein, quercitrin, kaempferol, formononetin, and 6-hydroxyflavanone that were previously identified by using UHPLC-ESI-MS/MS technique, were subjected to UHPLC quantification to determine the absolute amount of each standard in the most active extract. The result in Table 3 shows the metabolites content in FD *M. calabura* leaves extracted with 50% ethanol, ranging from 56.58 ± 0.28 to 650.01 ± 0.12 µg/mg of extract. The highest contents of metabolites in the extract were in the following order geniposide, daidzein, quercitrin, 6-hydroxyflavanone, kaempferol, and formononetin.

Each of these metabolites may contribute to the anti-hyperglycemic effect of *M. calabura* leaves. For example, geniposide plays an important role in reducing glycogenolysis process through impairment of hepatic glycogen phosphorylase and glucose-6-phosphatase activity, as well as their protein expression in high fat diet-streptozotocin (STZ) induced diabetic mice [18]. On the other hand, daidzein helps to increase the mRNA level in the β-cells to exert more insulin production, as well as restoring the glucose metabolic enzyme activities in daidzein-supplemented non-obese diabetic mice [19]. In addition, one of the quercetin derivatives has proven to have a protective effect on the intact β-cells of the islets of Langerhans by inhibiting lipid peroxidation, as well as its ability to scavenge free radicals from STZ-induced oxidative stress in STZ-induced diabetic rats [20]. Although no research has been conducted regarding the proficiency of 6-hydroxyflavanone on directly reducing the blood glucose level, there are some studies that have demonstrated the electron donating ability of 6-hydroxyflavanone to scavenge free radicals induced by 2,2-diphenyl-1-picrylhydrazyl in in vitro antioxidant study [21,59]. Besides that, kaempferol, another flavonoid-type compound that was identified and quantified, also reportedly shows hypoglycemic effect by lowering the levels of glycoprotein in the liver and increment of insulin production, as well as enhancement of glucose utilization in STZ-induced rats [22]. Formononetin helps in the activation of peroxisome proliferator-activated receptor (PPAR-γ) genes in liver, which, in turn, regulates the blood glucose level, the cell surface receptor protein (FAS) may not induce the cleavage of SREBP-1C site in stimulating lipid synthesis due to an antagonistic interaction between formononetin and PPAR-γ genes [23]. Therefore, these identified metabolites were quantified based on its positive attributes in lowering the blood glucose level, either through in vivo or in vitro study, that could potentially influenced the hypoglycemic aptitude of *M. calabura* leaves.

## 3. Materials and Methods

### 3.1. Chemicals and Reagents

LCMS grade formic acid, methanol, acetonitrile, water, dimethyl sulfoxide (DMSO), sodium phosphate buffer, and absolute ethanol were supplied by Thermo Fisher Scientific (Waltham, MA, USA). Other chemicals including p-nitrophenyl-α-D-glucopyranose (PNPG), α-glucosidase enzyme, α-amylase enzyme, glycine, 3,5-dinitrosalicylic acid (DNS), potato starch, and sodium phosphate buffered saline (PBS) were purchased from Sigma Aldrich (St. Louis, MO, USA). All the standard compounds including acarbose, quercetin, daidzein, quercitrin, kaempferol, formononetin, 6-hydroxyflavanone, and geniposide were purchased from Acros Organics (Geel, Belgium) with >98% purity.

### 3.2. Plant Materials

The fresh *M. calabura* were harvested from Universiti Putra Malaysia’s Mosque. Number of voucher specimen (SK 3345/18) was deposited by a botanist (Dr. Mohd Firdaus Ismail) in the herbarium of Institute of Bioscience, Universiti Putra Malaysia. The leaves of five different trees of *M. calabura* were harvested and separated into three drying processes, namely oven-drying (OD), air-drying (AD), and freeze-drying (FD). The fresh leaves were assigned at 40 °C in a convention oven (Protech, Seri Kembangan, Malaysia), for a duration of 10 days for OD processed leaves. On the other hand, for the AD sample, the leaves were dried at 37 °C for the duration of 2 weeks. As for the FD sample, the leaves were stored for 4 days in −80 °C freezer (Haier, Surrey, UK) prior to 3 days FD (LabConco, Kansas City, MO, USA). All samples were confirmed to absolute dryness, as their constant weight was achieved before grinding process started. The ground samples were then stored at 2–7 °C in airtight container until further analysis. Total 5 g of each ground leaves was soaked and sonicated in 100 mL of 0, 50, and 100% ethanol:water ratio, respectively, for 60 min by using ultrasonic bath sonicator (Kudos, Shanghai, China). The Whatman filter paper no. 1 was used to filter the mixture and the filtered mixture was concentrated under controlled temperature at 40 °C by using a rotatory evaporator (Buchi LaboratoriumsTechnik, Flawil, Switzerland). To ensure complete dryness, all extracts were then lyophilized in a freeze dryer (LabConco, Kansas City, MO, USA) and subsequently stored at −20 °C until further analysis.

### 3.3. In Vitro Anti-Diabetic Activity

#### 3.3.1. α-Glucosidase Inhibition Assay

The α-glucosidase inhibition activity assay has been conducted according to the demonstrated method with modifications [60]. The PNPG in 50 mM phosphate buffer (pH 6.5) has been used as the substrate, which was comparable to the intestinal fluid. Next, sample extracts were prepared at 200 μg/mL and 6 serial dilutions were done. In the 96-well plate, a reaction consisting of 130 μL of 30 mM phosphate buffer, 10 μL of enzyme, and 10 μL of extracts was incubated at 37 °C for 5 min. Then, 50 μL of substrate was added in the reaction and was further incubated at 37 °C for another 15 min. Next, 50 μL of 2 M glycine (pH 10) was then added to stop the reaction. The absorbance was measured by using a spectrophotometer (Spectramax PLUS, San Jose, CA, USA) at 405 nm wavelength. The α-glucosidase inhibitory activity was calculated using the equation; [(An − As)/An] × 100%, where An is the difference in absorbance of the negative control and all the blanks, and As is the difference in absorbance of the sample and all the blanks. The α-glucosidase inhibitory activity was expressed as IC_50_ value (μg/mL) to represent *M. calabura* extract concentration that was needed to inhibit the enzyme activity by 50%. Quercetin was tested and used as positive control.

#### 3.3.2. α-Amylase Inhibition Assay

The α-amylase inhibition activity assay was carried out based on the demonstrated method with modifications [32]. In a 96-well plate, a reaction mixture containing 50 μL of 100 mM phosphate buffer at pH 6.8, 10 μL of 2 U/mL porcine α–amylase, and 20 μL of varying concentrations of the extract (0.04, 0.07, 0.14, 0.28, and 0.56 mg/mL) was incubated at 37 °C for 20 min. Then, 20 μL of substrate (1% starch dissolved in 100 mM phosphate buffer at pH 6.8) was added, and the mixture was further incubated at 37 °C for 30 min. The reaction was then stopped by adding 100 μL of the DNS color reagent and boiled for 10 min. The absorbance was measured by using a spectrophotometer (Spectramax PLUS, San Jose, CA, USA) at 540 nm wavelength. The α-amylase inhibitory activity has been calculated using the equation; [(An − As)/An] × 100%, where An is the difference in absorbance of the negative control and all the blanks, and As is the difference in absorbance of the sample and all the blanks. The α-amylase inhibitory activity was expressed as IC_50_ (μg/mL). Acarbose was tested and employed as a positive control in each plate of α-amylase activity.

### 3.4. UHPLC-ESI-MS/MS Analysis

The sample preparation for UHPLC-ESI-MS/MS analysis was prepared based on the reported method with modifications [36]. The most active extract (4 mg) was dissolved in 2 mL of LCMS-grade methanol and filtered through a 0.22 µm filter prior to 20.0 µL injection to the UHPLC-ESI-MS/MS analysis. The analysis was performed using an Dionex Ultimate 3000 UHPLC coupled with Q Exactive™ Focus Orbitrap mass spectrometer (Thermo Fisher Scientific, Bremen, Germany) equipped with a binary pump, vacuum degasser, temperature-controlled autosampler, diode array detector (200–600 nm range; 5 nm bandwidth), and a heated electrospray ionization source. The separation was conducted on a Waters Acquity UPLC HSS T3 column (1.8 µm, 2.1 × 100 mm) (Waters Corp, Milford, MA, USA). The gradient solvent system was then commenced from 95:5–0:100 (*v*/*v*) of water with 0.1% formic acid: acetonitrile with 0.1% formic acid over 39 min, with a flow rate of 0.25 mL/min. Negative and positive ion modes were acquired and recorded on Q Exactive™ Focus Orbitrap mass spectrometer (Thermo Fisher Scientific, Bremen, Germany). The MSn analytical conditions were as follows: spray volt-pressure −3.6 kV; equipment temperature, 37.8 °C; capillary temperature, 300 °C; auxiliary gas at 8 units; sheath gas at 50 units; scan range *m/z* 150–1200, data acquisition frequency at 12 Hz, and collision-induced dissociation energy was adjusted to 30%. The data was recorded and processed using the Thermo Xcalibur Qual Browser software 4.0 (Thermo Fisher Scientific, Bremen, Germany).

### 3.5. Absolute Quantification from FD Leaves Extracted with 50% Ethanol by UHPLC

The most active extract was also subjected to absolute quantification for several phenolics named daidzein, quercitrin, kaempferol, formononetin, and 6-hydroxyflavanone, as well as a terpene glycoside, geniposide. The sample preparation for UHPLC analysis was according to the reported method with slight adjustment [36]. The active extract (10 mg) of the *M. calabura* was dissolved in 2 mL of LCMS-grade methanol and filtered through a 0.22 µm filter prior to 2.0 µL injection to the UHPLC analysis. The analysis was performed using a Waters Acquity UHPLC (Waters Corp, Milford, MA, USA) equipped with a binary pump, vacuum degasser, temperature-controlled autosampler, column heater, and 2998 photodiode array (PDA) detector (200–600 nm range; 5 nm bandwidth). The separation was conducted on a Waters Acquity UPLC HSS T3 column (1.8 µm, 2.1 × 150 mm) (Waters Corp, Milfor, MA, USA) with the same gradient solvent system mentioned in profiling section, where the gradient solvent system was commenced from 95:5–0:100 (*v*/*v*) of water with 0.1% FA:acetonitrile with 0.1% FA over 39 min with a flow rate of 0.25 mL/min. The detection wavelength was set at 254 nm. The data was evaluated and processed by using the Waters Empower Chromatography Data System software. The metabolite contents in the plant extract were calculated, based on the area under the peak, and expressed as µg/mg of plant extract.

The quantification method was validated according to International Council of Harmonization (ICH) tripartite guidelines [61] based on the following criteria:

- Specificity: the retention time of the standards and extract are complementary with no contaminants or impurities detected in the eluent, with the equal volume (2.0 µL) of sample, standards, and solvent injected into the chromatography system.
- Repeatability precision: all the relative standard deviation (RSD) were <2%, indicating high precision. The repeatability precision test was acquired by three-times injections at three concentration levels (5, 20, and 40 µg/mL) for each standard (daidzein, quercitrin, kaempferol, formononetin, 6-hydroxyflavanone, and geniposide).
- Linearity and range: the calibration curve was obtained by three data points at 5, 20, and 40 µg/mL. Each calibration curve was determined by the averaging triplicate value of each concentration. Table 4 shows the concentration range, regression equation, and correlation coefficient (R^2^).
- Limit of detection (LOD) and limit of quantification (LOQ) were determined by calculating the signal:noise ratio, established at 3.3:1 (LOD) and 10:1 (LOQ), based on the calibration curve with the following formula:
  LOD = (SD × 3.3)/M
  LOQ = (SD × 10)/M
  where SD is the standard deviation of the response, and M is the slope of calibration curve. Table 4 demonstrates the LOD and LOQ value for the six tested standards.

### 3.6. Statistical Analysis

The results of the five biological replicates were expressed in mean ± standard deviation. The statistical analysis was done by using Minitab software (Version 16, Minitab Inc, State College, PA, USA), while the significant difference in the results was determined by analysis of variance (ANOVA) with post hoc Tukey pairwise multiple-comparisons test (*p* < 0.05, significant).

## 4. Conclusions

In conclusion, *M. calabura* leaves, extracted with 50% ethanol and dried by using FD method, were revealed to be the best condition to extract the anti-diabetic metabolites. A total of sixty-one compounds were tentatively identified in the active extract by using UHPLC-ESI-MS/MS technique, including five catechin derivatives, five types of kaempferol derivatives, three types of quercetin derivatives, one apigenin derivative compound, three types of luteolin derivatives, seven types of daidzein derivatives, nineteen types of other flavonoids, two types of anthocyanin compounds, two types of chalcone compounds, six types of quinone derivatives, two types of lactones derivatives, two types of alkaloid derivatives, two types of sugar, one terpene glycoside, and one ellagitannins derivative. The highest contents of metabolites in the active extract were in the following order: geniposide, daidzein, quercitrin, 6-hydroxyflavanone, kaempferol, and formononetin. Hence, this study suggests that, with the incorporation of the FD method, *M. calabura* leaves when extracted with 50% ethanol, can play a significant role as a potential for the development of naturally derived herbal medicinal components that not only help to inhibit diabetes related complications but also impedes toxic side effects of synthetic anti-diabetic drugs.

## Figures and Tables

**Table 1:** α-Glucosidase and α-amylase inhibitory activity of *M.* *calabura* leaves dried with oven-drying (OD), air-drying (AD), and freeze-drying (FD) and extracted with 0, 50, and 100% ethanol.

Drying Method	Ethanol:Water Ratio	α-Glucosidase Inhibitory Assay IC_50_ (µg /mL)	α-Amylase Inhibitory Assay IC_50_ (µg /mL)
OD	100	1.13 ± 0.13 ^Ba^	59.39 ± 2.47 ^Ba^
	50	0.81 ± 0.09 ^Ca^	45.77 ± 2.46 ^Ca^
	0	2.76 ± 0.09 ^Aa^	105.95 ± 1.57 ^Ac^
AD	100	1.07 ± 0.06 ^Ba^	53.34 ± 1.64 ^Bb^
	50	0.59 ± 0.14 ^Bb^	35.32 ± 2.35 ^Cb^
	0	2.41 ± 1.00 ^Aa^	114.43 ± 2.22 ^Ab^
FD	100	0.65 ± 0.04 ^Bb^	23.84 ± 1.85 ^Bc^
	50	0.46 ± 0.05 ^Bb^	26.39 ± 3.93 ^Bc^
	0	2.01 ± 0.86 ^Aa^	185.17 ± 2.11 ^Aa^
Standard	Quercetin	2.15 ± 0.26	−
	Acarbose	−	0.68 ± 0.14

The IC_50_ values of five biological replicates are expressed as means ± standard deviation. The uppercase letter is used to demonstrate the different ethanol:water ratio for the same drying process, while the lowercase letter is used to demonstrate the varied drying process for the same ethanol:water ratio. The different superscript letters represent significant differences at *p* < 0.05 between samples.

**Table 3:** Concentration of metabolites in *M. calabura* FD leaves extracted with 50% ethanol.

Metabolites	Concentration (µg/mg of Extract)
Geniposide	650.01 ± 0.12
Daidzein	231.65 ± 0.31
Quercitrin	223.24 ± 0.59
Kaempferol	75.22 ± 0.72
Formononetin	56.58 ± 0.28
6-Hydroxyflavanone	196.43 ± 0.28

**Table 4:** Linearity, LOD, and LOQ values of the UHPLC method for the standard compounds.

Standards	Concentration Range (µg/mL)	Regression Equation	Correlation Coefficient (R^2^)	LOD (µg/mL)	LOQ (µg/mL)
Daidzein	5.1592–40.2551	Y = 2.67 × 10^4^X − 2.33 × 10^4^	0.999	0.10	0.30
Quercitrin	4.7603–39.9357	Y = 1.83 × 10^4^X − 2.45 × 10^4^	0.999	0.04	0.11
Kaempferol	5.4330–40.6827	Y = 1.82 × 10^4^X − 3.33 × 10^4^	0.999	0.17	0.52
Formononetin	5.1813–40.1937	Y = 2.05 × 10^4^X − 1.45 × 10^4^	0.999	0.19	0.56
6-Hydroxyflavanone	5.1507–40.1779	Y = 1.58 × 10^4^X− 4.56 × 10^4^	0.999	0.03	0.10
Geniposide	5.7511~40.7790	Y = 4.16 × 10^3^X − 6.01 × 10^3^	0.999	0.32	0.96

## Data Availability

The data are available within this article.

## Sample Availability

Samples of the compounds are not available from the authors.
